# Supplementary material for: Steering magnonic dynamics and permeability at exceptional points in a parity–time symmetric waveguide
Source: Nat Commun. 2020 Nov 9;11:5663. doi: 10.1038/s41467-020-19431-3 (PMC7652947; doi:10.1038/s41467-020-19431-3)
Supplement: Supplementary file 1 — Supplementary Information [file 41467_2020_19431_MOESM1_ESM.pdf]

Supporting Information: Steering magnonic dynamics and permeability at exceptional points in a parity-time symmetric waveguide

Xi-guang Wang,[†,‡] Guang-hua Guo,[†] and Jamal Berakdar[*,‡]

† *School of Physics and Electronics, Central South University, Changsha 410083, China*

‡*Institut für Physik, Martin-Luther Universität Halle-Wittenberg, D-06120 Halle/Saale, Germany*

E-mail: jamal.berakdar@physik.uni-halle.de

Technical details of the numerical simulations

The LLG equation is numerically solved employing a fifth-order Runge-Kutta scheme with a fixed time step of 0.2 ps. We discretize the two coupled waveguides with a mesh cell size of 4 nm. In the simulation, we first let the magnetization relax to a stationary state ($+y$ direction), and then add the spin wave excitation in WG1 or WG2. For the spin wave excitation we apply a microwave magnetic field $h(t) = h_{\rm a}\mathbf{x}sin(2\pi ft)$ with an amplitude $h_{\rm a} = 100$ A/m and a variable frequency. The field is applied locally to the region of the waveguides that we take as the origin $x = 0$. The spin waves propagate along the $x-$ direction. The spin wave amplitude is related to the oscillation amplitudes of the x component $m_{\rm x}(x,t)$ and z component $m_{\rm z}(x,t)$ of the magnetization. To suppress the spin-wave reflection from geometric boundaries, a sufficiently large (40μm) extension along x is adopted in the simulation.

Dynamic magnetic permeability in separated waveguides

To prove that the system exhibits a PT-symmetric behavior we calculate the transverse dynamic permeability in two separate waveguides (meaning the coupling between two waveguides are dropped). The accumulated spin density polarizations at WG1 and WG2 are $\mathbf{T}_1 = \mathbf{y}$ and $\mathbf{T}_2 = -\mathbf{y}$. Around the equilibrium state ($\mathbf{m}_0 = \mathbf{y}$), we linearize the LLG equation and obtain

$$\begin{aligned} \dot{m}_{\mathrm{x}} - \alpha\dot{m}_{\mathrm{z}} &= \omega_{\mathrm{H}} m_{\mathrm{z}} - \omega_{\mathrm{ex}}\partial_{\mathrm{x}}^2 m_{\mathrm{z}} \pm \omega_{\mathrm{c}} m_{\mathrm{x}} - \gamma h_{\mathrm{z}}, \\ \dot{m}_{\mathrm{z}} + \alpha\dot{m}_{\mathrm{x}} &= -\omega_{\mathrm{H}} m_{\mathrm{x}} + \omega_{\mathrm{ex}}\partial_{\mathrm{x}}^2 m_{\mathrm{x}} \pm \omega_{\mathrm{c}} m_{\mathrm{z}} + \gamma h_{\mathrm{x}}. \end{aligned} \tag{1}$$

Here, $\omega_{\mathrm{H}} = \gamma H_0$, $\omega_{\mathrm{c}} = \gamma c_{\mathrm{J}}$, $\omega_{\mathrm{ex}} = \frac{2\gamma A_{\mathrm{ex}}}{\mu_0 M_{\mathrm{s}}}$, $h_{\mathrm{x,z}}$ is the microwave magnetic field acting on the magnetization. $+\omega_{\mathrm{c}}$ and $-\omega_{\mathrm{c}}$ correspond to WG1 and WG2, respectively.

By defining the Fourier transforms $\widetilde{g}(k_{\mathrm{x}}, \omega) = \int dx e^{ik_{\mathrm{x}}x} \int dt e^{i\omega t} g(x,t)$ and $g(x,t) = \frac{1}{l}\int dk_{\mathrm{x}} e^{-ik_{\mathrm{x}}x} \int \frac{d\omega}{2\pi} e^{-i\omega t}\widetilde{g}(k_{\mathrm{x}}, \omega)$, where l is the length of the waveguides, Eq. (1) reads $\widetilde{m}_{\mathrm{p}} = \sum_{\mathrm{pq}} \chi_{\mathrm{pq}}\gamma\widetilde{h}_{\mathrm{q}}$ with $p, q = x, z$, and the transverse dynamic magnetic susceptibility $\chi^{1,2}(k_{\mathrm{x}}, \omega)$ for WG1 and WG2 are

$$\chi^{1,2} = \frac{1}{(\omega_{\mathrm{k}} - i\alpha\omega)^2 + (-i\omega \pm \omega_{\mathrm{c}})^2} \begin{pmatrix} \omega_{\mathrm{k}} - i\alpha\omega & -(-i\omega \pm \omega_{\mathrm{c}}) \\ -i\omega \pm \omega_{\mathrm{c}} & \omega_{\mathrm{k}} - i\alpha\omega \end{pmatrix}. \tag{2}$$

Here, $+\omega_{\mathrm{c}}$ and $-\omega_{\mathrm{c}}$ correspond respectively to WG1 and WG2, and $\omega_{\mathrm{k}} = \omega_{\mathrm{H}} + \omega_{\mathrm{ex}} k_{\mathrm{x}}^2$. Neglecting the influence of α, the symmetry of the dynamic magnetic susceptibilities of the two waveguides is expressed as $\chi^1_{\mathrm{xx,zz}} = (\chi^2_{\mathrm{xx,zz}})^*$ and $\chi^1_{\mathrm{xz,zx}} = -(\chi^2_{\mathrm{xz,zx}})^*$.

The effects of residual magnetic damping

From the above dynamic magnetic susceptibilities, we extract the effective damping constant $\alpha^{1,2}_{\mathrm{eff}} = \alpha \pm \omega_{\mathrm{c}}/\omega_{\mathrm{k}}$ in the two uncoupled waveguides. With the parameters used in the main

article, we have $\omega_c/\omega_k = 0.17$ under $k_x = 0.1$ nm^{-1} and $\omega_c = \kappa = 22.1$ GHz. The Gilbert damping constant $\alpha = 0.004$ is subsidiary in comparison to ω_c/ω_k. To justify the inequalities involving the α related terms in the main article (i.e., $\omega_0 \gg \alpha\omega_J$ and $\omega_J \gg \alpha\omega_0$), we note that for $\omega_J = \kappa = 22.1$ GHz and $\omega_0 = 149$ GHz under $k_x = 0.1$ nm^{-1}, we have $\alpha\omega_J = 0.09$ GHz and $\alpha\omega_0 = 0.6$ GHz with $\alpha = 0.004$.

For a further demonstration of the influence of damping, we calculate the two eigenvalues assuming a larger $\alpha = 0.08$. The results are displayed in Fig. 1. Below the threshold $\omega_J/\kappa < 1$, a slight difference between the two imaginary parts of the eigenvalues is identified. At the exceptional point $\omega_J/\kappa = 1$, the two eigenvalues also become identical. Above the exception point $\omega_J/\kappa > 1$, the real parts of the two eigenvalues are separated by a sufficiently large damping term.

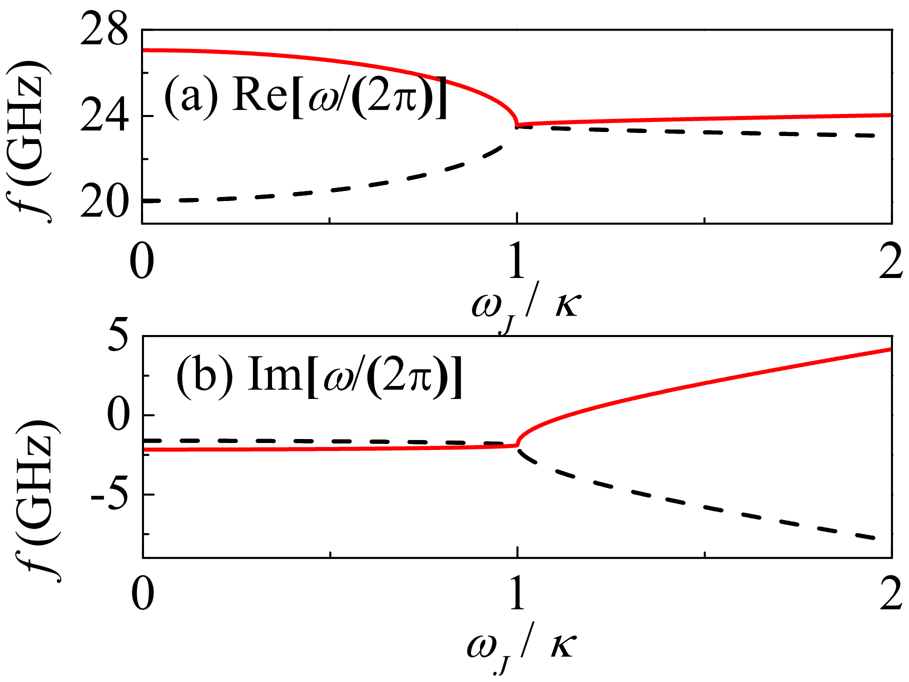

Figure 1: For $\alpha = 0.08$, (a) real and (b) imaginary parts of the two eigenvalues ω as a function of ω_J when $k_x = 0.1$ nm^{-1}.

Influence of magnetic damping on the PT symmetry behavior of the waveguides

In this section, we provide more details on the interplay between residual magnetic damping and the PT symmetry-related magnonic properties of the waveguides. Rewriting Eq. (2) of the main text in the form of a vector equation, $i\frac{\partial \boldsymbol{\psi}}{\partial t} = \hat{H}\boldsymbol{\psi}$, where $\boldsymbol{\psi} = (\psi_1, \psi_2)$, and the 2×2 Hamiltonian $\hat{H}$ is non-Hermitian and has the explicit form

$$\hat{H} = \begin{pmatrix} (\omega_0 - \alpha\omega_{\mathrm{J}}) - i(\omega_{\mathrm{J}} + \alpha\omega_0) & -q \\ -q & (\omega_0 + \alpha\omega_{\mathrm{J}}) + i(\omega_{\mathrm{J}} - \alpha\omega_0) \end{pmatrix}. \tag{3}$$

The eigenvalues and eigenvectors are generally complex,

$$\begin{aligned} \omega_{\pm} &= (1 - i\alpha)\omega_0 \pm \sqrt{q^2 - \omega_{\mathrm{J}}^2 + 2i\alpha\omega_{\mathrm{J}}^2 + \alpha^2\omega_{\mathrm{J}}^2}, \\ \boldsymbol{\psi}_{\pm} &= (\frac{(i+\alpha)\omega_{\mathrm{J}} \mp \sqrt{q^2 - \omega_{\mathrm{J}}^2 + 2i\alpha\omega_{\mathrm{J}}^2 + \alpha^2\omega_{\mathrm{J}}^2}}{q}, 1). \end{aligned} \tag{4}$$

In the limit $\alpha \to 0$ ($q \equiv \kappa$), the two different eigenvalues are always real and different $\omega_{\pm} = \omega_0 \pm \sqrt{\kappa^2 - \omega_{\mathrm{J}}^2}$ in the PT-symmetric regime below the gain/loss-balance threshold $\omega_{\mathrm{J}}/\kappa < 1$, and the eigenvectors can be given by $\boldsymbol{\psi}_{\pm} = (\mp \exp(\mp i\theta), 1)$, where $\sin(\theta) = \omega_{\mathrm{J}}/\kappa$. At $\omega_{\mathrm{J}}/\kappa = 1$, the eigenvalues and the eigenvectors of this Hamiltonian coalesce at $\omega_{\pm} = \omega_0$ and $\boldsymbol{\psi}_{\pm} = (i, 1)$, which is the hallmark of the exceptional point (EP). Above the EP ($\omega_{\mathrm{J}}/\kappa > 1$), the eigenvalues turn complex ($\omega_0 \pm i\sqrt{\omega_{\mathrm{J}}^2 - \kappa^2}$), where the two real parts of $\omega_{\pm}$ are both ω_0, and the two imaginary parts are separated, and the eigenvectors are $\boldsymbol{\psi}_{\pm} = (i\frac{\omega_{\mathrm{J}} \mp \sqrt{\omega_{\mathrm{J}}^2 - \kappa^2}}{\kappa}, 1)$. These changes in eigenmodes bring about a non-reciprocal response and power oscillations. Without SOT ($\omega_{\mathrm{J}} = 0$), the superposition of the two eigenmodes $\boldsymbol{\psi}_{\pm} = (\mp 1, 1)$ (spin waves modes in the two waveguides are antisymmetric and symmetric) leads to reciprocal wave propagation. The interference between the two modes leads to a periodic transfer of energy from one waveguide to the other, and the spin waves distributions obey the symmetry of

the two waveguides (see Fig. 1(d) in the main text). If ω_{J} increases but is still below the EP, the phase angle changes from the initial value $\theta = 0$ reaching eventually $\theta = \pi/2$ at the EP. In this range, the superposition of two asymmetric spin wave modes leads to the non-reciprocal wave propagation, where the spin wave distribution at the output end can be entirely different by exchanging the input from one waveguide to the other (Fig. 1(e) in the main text). At EP, the two spin waves modes coalesce to the same mode, and spin waves in the two waveguides travel simultaneously (Fig. 1(f) in the main text).

To understand the influence of damping, we expand Eq. (4) in series by neglecting α^2 and higher order terms (as α is usually much smaller than 1) which yields

$$\begin{aligned} \omega_{\pm} &= (\omega_0 \pm \sqrt{\kappa^2 - \omega_{\mathrm{J}}^2}) - i\alpha(\omega_0 \pm \sqrt{\kappa^2 - \omega_{\mathrm{J}}^2}), \\ \boldsymbol{\psi}_{\pm} &= (i\frac{\omega_{\mathrm{J}} \mp \sqrt{\omega_{\mathrm{J}}^2 - \kappa^2}}{\kappa}, 1). \end{aligned} \tag{5}$$

From the above equations, we infer that the two complex eigenvalues still merge at the same degenerate EP $\omega_{\mathrm{J}}/\kappa = 1$. The existence of EP in coupled dissipative dynamical systems has been discussed in Ref. [1]. Below EP ($\omega_{\mathrm{J}}/\kappa < 1$), the separation of the imaginary parts of the two eigenvalues is very weak due to the smallness of α. The separation suddenly becomes obvious above EP. Similarly, the real parts of $\omega_{\pm}$ are obviously distinct below EP, and their difference is very weak above EP. With $\alpha = 0.004$, the results in our main article confirm this conclusion. Furthermore, we find the α dependent term does not affect the eigen-vectors $\boldsymbol{\psi}_{\pm}$ of the two spinwaves modes. This shows that ω_{J} induces non-reciprocal propagation below EP and simultaneous propagation at EP, as shown in the main article. Also, we studied the case of a very large damping $\alpha = 0.08$. The results in Fig. 1 follow the above analysis.

Enhanced sensitivities

In the main text we reported on an enhanced sensitivity to slight changes in magnetic fields at EP. This feature is beneficial for sensing variations in the magnetic environment or

for increasing the magnetic response in photonic applications. To quantify this sensitivity increase we apply a perturbation $\epsilon_1\kappa$ and $\epsilon_2\kappa$ in WG1 and WG2, where the perturbations $\epsilon_{1,2} \ll 1$ negligibly affect the coupling. In this case, the perturbed waveguide equations become,

$$\begin{aligned} i\frac{\partial\psi_1}{\partial t} - [(\omega_1 - \alpha\omega_{\rm J}) - i(\omega_{\rm J} + \alpha\omega_1)]\psi_1 + q\psi_2 = 0, \\ i\frac{\partial\psi_2}{\partial t} - [(\omega_2 + \alpha\omega_{\rm J}) + i(\omega_{\rm J} - \alpha\omega_2)]\psi_2 + q\psi_1 = 0. \end{aligned} \tag{6}$$

Here, $\omega_1 = \omega_0 + \epsilon_1\kappa$ and $\omega_2 = \omega_0 + \epsilon_2\kappa$. Assuming the perturbation affects only WG1, at the EP $\omega_{\rm J} = \kappa$, the obtained eigenfrequencies can be expanded perturbatively using a Newton-Puiseux series[2] that begins with a square-root element, and the first three terms of this series are

$$\begin{aligned} &\omega_\pm \approx \kappa(c_0 \pm c_1\epsilon^{1/2} + c_2\epsilon), \\ &c_0 = (1 - i\alpha)\omega_0/\kappa, \\ &c_1 = (1 - i\alpha)e^{-i\pi/4}, \text{and } c_2 = (1 - i\alpha)/2. \end{aligned} \tag{7}$$

These expressions indicate that, the real parts of the eigenfrequencies bifurcate with a square-root dependence on the applied perturbation, i.e. $\mathrm{Re}[\omega_+ - \omega_-] = (1 - \alpha)\sqrt{2\epsilon}\kappa$.

Excitations of spin waves

To realize spin waves excitation experimentally, we suggest the structure shown in Fig. 2. Putting a stripe antenna perpendicular to x axis, the dynamic magnetic field from the injected microwave current excites locally propagating spin waves in one waveguide. The input spin waves propagate to the middle region in Fig. 2 where PT symmetry phase transition is effective, and the output spin wave strength can be pick up to the right end via conventional spin-wave detection techniques.

To circumvent difficulties in exciting spin waves with a large wave vector, one can combine the method proposed in Ref. [3]. Via microwave magnetic fields from antennas, large-wavelength spin waves are launched in the region with large internal effective magnetic

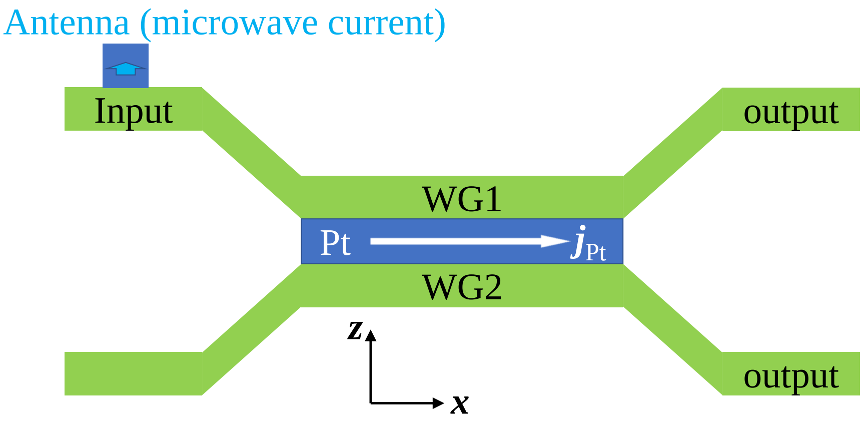

Figure 2: Schematics of the spin wave excitation of the PT symmetric coupled waveguides.

field. The wavelength becomes smaller when the wave crosses a region with a lower internal effective magnetic field. The different effective magnetic fields can be achieved by changing the geometry of the sample, attaching an exchange bias layer to the part of the magnetic layer, or covering the waveguide by a superconducting material (at the temperature below $T < T_c$). In addition, the emergent net ferroelectric polarization couples (through the magneto-electric coupling which mimics a dynamical DM interaction) to an external electric field. Thus, the applied nonuniform electric field is similar to a nonuniform DM term and leads to a particular type of torque called inhomogeneous electric torque.[4] The expression of the inhomogeneous electric torque is similar to the spin-transfer torque $l_{\mathrm{E}}\mathbf{m} \times (\mathbf{m} \times \mathbf{p}_{\mathrm{E}})$. The vector $\mathbf{p}_{\mathrm{E}} = \mathbf{x} \times \mathbf{e}_{\mathrm{i}}$, $\mathbf{e}_{\mathrm{i=x,y,z}}$ points to the direction of the electric field, and the electric field gradient $\partial_{\mathrm{x}} E_{\mathrm{i}}$ determines the coefficient $l_{\mathrm{E}} = -\gamma c_{\mathrm{E}} \partial_{\mathrm{x}} E_{\mathrm{i}}/(\mu_0 M_{\mathrm{s}})$. Therefore, the applied oscillating inhomogeneous electric field $E_{\mathrm{y}} = 0 (x < 0)$ and $E_{\mathrm{y}} = E_1(t)(x > 0)$, with $\mathbf{p_E} = \mathbf{z}$ at $x = 0$, leads to an oscillating electric torque which effectively excites the magnetization oscillation at $x = 0$ resulting in propagating spin waves. The inhomogeneous electric field can be realized by a combination of a uniform electric field and a normal metallic cap layer shielding the region $x < 0$.

Effects of varying the RKKY coupling strength

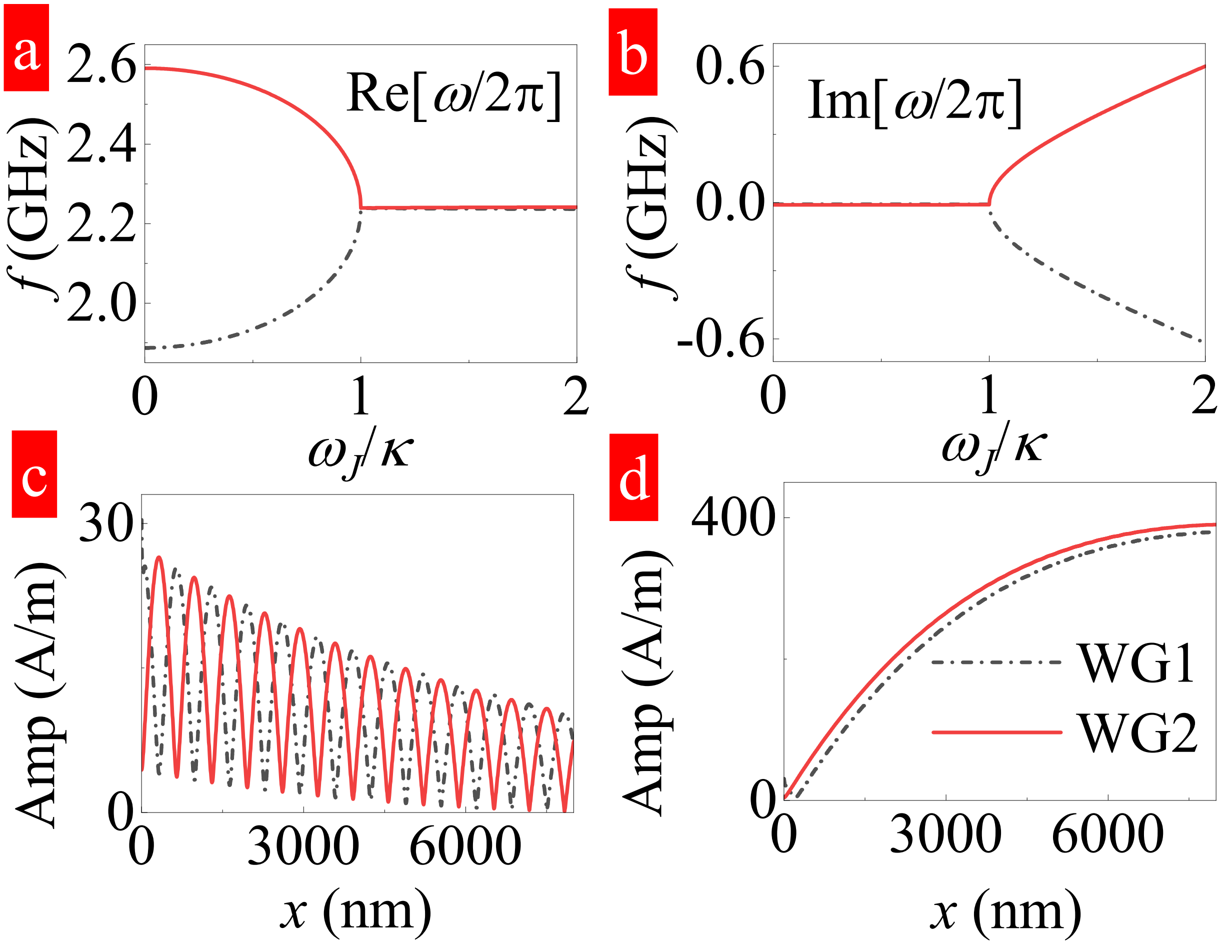

Figure 3: With smaller coupling strength $J_{\rm RKKY} = 9 \times 10^{-6}$J/m^2 and $H_0 = 2 \times 10^4$ A/m, (a) real and (b) imaginary parts of two eigenmode frequencies $f = \omega/(2\pi)$ as we scan $\omega_{\rm J}/\kappa$ at the wave vector $k_{\rm x} = 0.1$ nm^{-1}. (c-d) Spatial profiles of excited spin wave amplitudes for (c) $\omega_{\rm J} = 0$ and (d) EP $\omega_{\rm J} = \kappa$. Spin waves are excited by a local microwave field $h_0 \sin(\omega_{\rm h} t)$ ($h_0 = 100$ A/m and $\omega_{\rm h}/(2\pi) = 2.5$ GHz) at $x = 0$ in WG1.

Depending on the distance between the two ferromagnetic waveguides, the RKKY interaction $J_{\rm RKKY}$ which is responsible for the interlayer coupling can vary from positive to negative.[5] Let us consider situations with different $J_{\rm RKKY}$. We first consider a smaller positive $J_{\rm RKKY}$ which allows operating at a lower frequency. This is advantageous for an experimental realization, as the excitation of spin waves with high frequency is challenging. In Fig. 3, we use a smaller $J_{\rm RKKY} = 9 \times 10^{-6}$J/m^2 and an external magnetic field $H_0 = 2 \times 10^4$ A/m. Still, the EP is present at $\omega_{\rm J} = \kappa$, and the spin waves can travel simultaneously in

both waveguides at EP. With a smaller J_{RKKY} the spin wave has a frequency around 2 GHz, which is much smaller than the frequency studied in the main text (around 20 GHz).

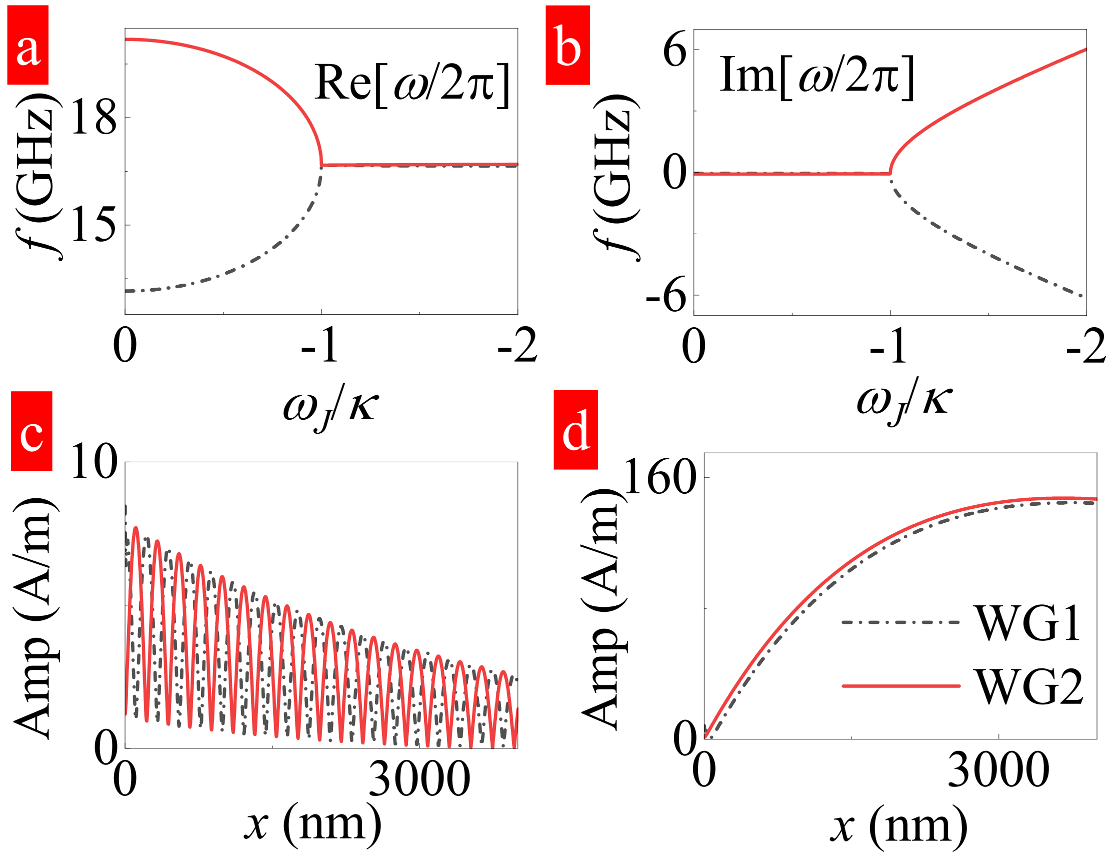

Figure 4: With negative coupling $J_{\mathrm{RKKY}} = -9 \times 10^{-5}\mathrm{J/m}^2$ and $H_0 = 2 \times 10^5$ A/m, (a) the real and (b) the imaginary parts of the two eigenmode frequencies $f = \omega/(2\pi)$ as we scan $\omega_{\mathrm{J}}/\kappa$ at the wave vector $k_{\mathrm{x}} = 0.1\ \mathrm{nm}^{-1}$. (c-d) Spatial profiles of excited spin wave amplitudes for (c) $\omega_{\mathrm{J}} = 0$ and (d) EP $\omega_{\mathrm{J}} = -\kappa$. Spin waves are excited by the local microwave field $h_0 \sin(\omega_{\mathrm{h}} t)$ ($h_0 = 100$ A/m and $\omega_{\mathrm{h}}/(2\pi) = 17$ GHz) at $x = 0$ in WG1.

We also considered the case of a negative $J_{\mathrm{RKKY}} = -9 \times 10^{-5}\mathrm{J/m}^2$. Without external magnetic fields, the stable configuration in waveguides' layers is antiferromagnetic. Applying an external magnetic field $H_0 = 2 \times 10^5$ A/m (the effective RKKY exchange field is around 1×10^5 A/m) and brings the waveguides into a stable parallel state. In this case, the spin wave dynamics follow the equations in the main text, and we observe the two eigenmodes merging at the EP $\omega_{\mathrm{J}}/\kappa = -1$, as shown in Fig. 4.

Coupled permalloy waveguides

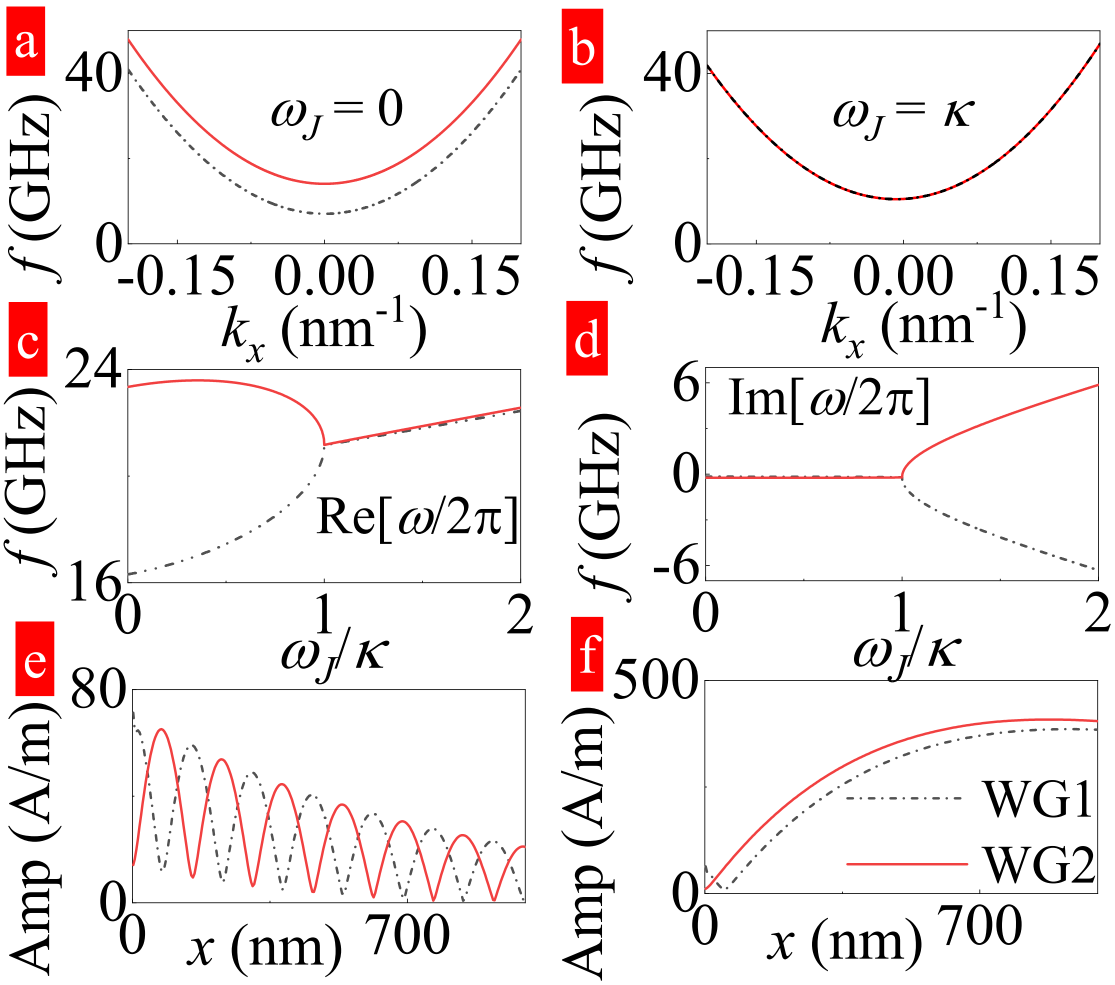

Figure 5: Permalloy waveguides are used. The material parameters are ($M_s = 8.6 \times 10^5$ A/m, $A_{ex} = 13 \times 10^{-12}$ J/m, $\alpha = 0.01$, $J_{RKKY} = 4 \times 10^{-4}$ J/m^2, $P = 0.4$ and $T\theta_{SH} = 0.07$), (a-b) Merging of the two magnon modes dispersion (a, $\omega_J = 0$) when approaching the EP (b, $\omega_J = \kappa$). (c) Real and (d) imaginary parts of two eigenmode frequencies $f = \omega/(2\pi)$ as we scan ω_J/κ at the wave vector $k_x = 0.1$ nm^{-1}. (e-f) The real-space distribution of the excited spinwave amplitudes for (e) $\omega_J = 0$ and (f) EP $\omega_J = \kappa$. spin waves are excited by the local microwave field $h_0 \sin(\omega_h t)$ ($h_0 = 100$ A/m and $\omega_h/(2\pi) = 20$ GHz) at $x = 0$ in WG1.

In this section, we extend our study to coupled metallic ferromagnetic waveguides, in the calculations we use permalloy. The electric current flowing in the Pt spacer causes spin orbit torques (SOTs) on the two coupled waveguides $\frac{\gamma c_J}{M_s}\mathbf{M}_p \times \mathbf{T}_p \times \mathbf{M}_p$.[6,7] In addition, the electric current flows in the metallic ferromagnetic waveguides possibly causing an in-plane spin transfer torque (STT) $b_J \partial_x \mathbf{M}_p$.[8] Here, $c_J = T\theta_{SH}\frac{\hbar\, J_e}{2\mu_0 e\, t_p M_s}$ and $b_J = \frac{g\mu_B P J_e}{2eM_s}$, g is

the Landé factor, μ_{B} is the Bohr's magneton, P is the spin-polarization efficiency, e is the electron charge, t_{p} is the thickness of pth waveguide, θ_{SH} is the spin Hall angle,and M_{s} is the saturation magnetization. Including the SOT and STT in the LLG equation, and following the same derivation as in the main text, we obtain the eigenfrequencies for the optical and acoustic magnon modes,

$$\omega = (1 - i\alpha)(\omega_0 \pm \sqrt{\kappa^2 - \omega_{\mathrm{J}}^2}) \tag{8}$$

Here, $\kappa = \frac{\gamma J_{\mathrm{RKKY}}}{(1+\alpha^2)\mu_0 M_{\mathrm{s}} t_{\mathrm{p}}}$, $\omega_{\mathrm{J}} = \frac{\gamma c_{\mathrm{J}}}{1+\alpha^2}$ and $\omega_0 = \frac{\gamma}{1+\alpha^2}(H_0 + \frac{2A_{\mathrm{ex}}}{\mu_0 M_{\mathrm{s}}} k_{\mathrm{x}}^2 + \frac{J_{\mathrm{RKKY}}}{\mu_0 M_{\mathrm{s}} t_{\mathrm{p}}}) + \frac{b_{\mathrm{J}} k_{\mathrm{x}}}{1+\alpha^2}$. From the above equation, one can see that, in the metallic coupled waveguides, two magnons modes still merge at the EP $\omega_{\mathrm{J}} = \kappa$. The existence of the in-plane spin transfer torque (b_{J} term) leads a weak asymmetry in the magnon dispersion, as shown by the calculations in Fig. 5. Above EP, the increase of b_{J} can further enhance ω (Fig. 5(c-d)). The existence of EP is further evidenced by inspecting the magnon propagation in Fig. 5(e-f).

Influence of the dipole-dipole interaction

For an experimental realization it is important to address the role of the dipole-dipole interaction and how it may influence PT-symmetry-related effects. To this end we conducted micromagnetic simulations and analytical calculations and summarize the results in this section. Generally, for coupled nano-strip waveguides with a finite size accounting for the influences of demagnetization field, we find that the dipole-dipole interaction between the magnons in the two waveguides slightly increases the value of EP in the low frequency range. The main conclusions concerning the SOT driven PT-symmetry-behavior are however unaltered.

For a numerical implementation let us adopt a similar model, as discussed in the main text. Two stripe waveguides are coupled via the RKKY interaction and the dipole-dipole interaction across the non-magnetic spacer. The demagenetization field enters the effective

field of LLG equation as,

$$\mathbf{H}_{\text{demag}}(\mathbf{r}) = -\frac{M_{\text{s}}}{4\pi}\int_{\text{V}} \nabla\nabla' \frac{1}{|\mathbf{r}-\mathbf{r}'|}\mathbf{m}(\mathbf{r}')d\mathbf{r}'. \tag{9}$$

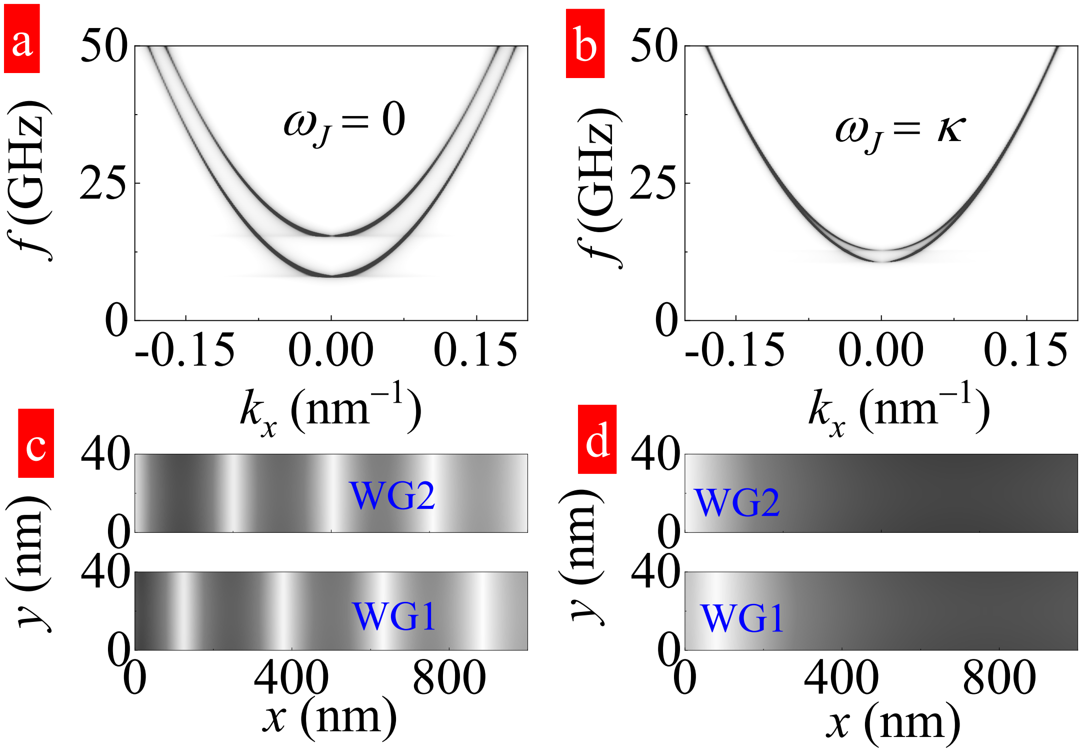

Figure 6: (a-b) Simulated spin wave dispersion relation in the coupled waveguides for (a) $\omega_{\text{J}} = 0$ and (b) $\omega_{\text{J}} = \kappa$. (c-d) The spatial profiles of the propagating spin wave amplitude (c) $\omega_{\text{J}} = 0$ and (d) $\omega_{\text{J}} = \kappa$. The spin waves at 30 GHz are locally excited by a local microwave field at the left end in WG1. The color change from white to black corresponds to a linear amplitude change ranging from 0 to maximum of spin wave amplitude.

In micromagnetic simulations based on LLG equation with dipole-dipole interaction Eq. (9), the two stripes have the same size of $x \times y \times z = 4\mu\text{m} \times 40\text{nm} \times 4\text{nm}$ with a unit simulation cell of $4\text{nm} \times 4\text{nm} \times 4\text{nm}$. The thickness of the spacer is also 4 nm. The material parameters are those from the main text with $\alpha = 0.01$. For the numerical integration of the LLG equation, we utilize the Dormand-Prince method (RK45) with a fixed time step of 0.2 ps. In the simulation, we first let the magnetization relax to the stationary state (which is along the y axis), and then add the spin wave excitation on top of the ground state. For exciting

spin waves over a wide frequency range, the field pulse $\mathbf{h}(t) = h_a \sin(2\pi f_H t)/(2\pi f_H t)\mathbf{x}$ with $h_a = 1$ T and a cutoff frequency of $f_H = 50$ GHz is applied locally to the left end of the waveguides. We analyze the fluctuation statistics extracted from each cell m_x during 20 ns with a time step of 5 ps. The obtained frequency resolution of the spectrum is 0.05 GHz. For calculating the spin wave dispersion relations we use a two-dimensional fast Fourier transformation (FFT) $m_{p,x}(k_x, f) = \frac{1}{N_y}\sum_{i=1}^{N_y} F_2[m_{p,x}(x, y_i, t)]$. Here, y_i is the i-th cell along y axis, and N_y is the total cell number along y axis.

The results for the spin wave dispersion relation in the coupled waveguides are shown in Figs. 6(a-b). The combination of RKKY coupling and the dipolar coupling between the two waveguides leads to the formation of the two spinwave modes (acoustic and optical modes). The dispersion of the two modes clearly splits when $\omega_J = \gamma c_J = 0$. At the lower frequencies, the gaps between the two modes are larger as the dipolar coupling becomes more important. Applying SOT at EP $\omega_J = \kappa = \gamma\frac{J_{RKKY}}{\mu_0 M_s t_p}$, the two spin wave modes become identical in the high frequency range, while the gap between the two spin wave modes in lower frequency range becomes much smaller. Also, we analyzed the spin wave transmission. Without SOT ($\omega_J = 0$), the spin waves injected at one end in one of the two waveguides oscillates between WG1 and WG2 during propagation. Applying SOT at EP $\omega_J = \kappa$, we find the spin waves travel simultaneously in both waveguides. All results here confirm our findings about PT symmetry and the presence of EP in coupled waveguides the main text. Thus, we may conclude that for the investigated case the dipole-dipole interaction does not alter qualitatively the predicted PT-symmetry-related phenomena.

To provide more details on the magnon propagation in the coupled waveguides with the dipole-dipole interaction, we develop a simple analytical model. The magnetization vectors with small derivations in the two waveguides ($p = 1, 2$) have the form $\mathbf{m}_p(r,t) = \mathbf{m}_{0,p} + \mathbf{m}_{s,p}e^{i(\mathbf{k}_s\cdot\mathbf{r}-\omega t)}$. Here, $\mathbf{m}_{0,p} = \mathbf{y}$ is the static equilibrium magnetization parallel to y axis, and the small deviation from the equilibrium is $\mathbf{m}_{s,p} = (\delta m_{x,p}, 0, \delta m_{z,p})$ with $\delta m_{x(z),p} \ll 1$. The wave vector $\mathbf{k}_s$ is the sum of the in-plane wave vector $\mathbf{k} = k_x\mathbf{x} + k_y\mathbf{y}$ and the perpendicular

(to the x-y plane) wave vector k_z. Then, from the Fourier representation of the linearized LLG equation, we derive the following expression for the spin wave,

$$-i\omega \mathbf{m}_{\mathrm{s,p}} = \mathbf{m}_{0,\mathrm{p}} \times \sum_{\mathrm{q}} \hat{\Omega}_{\mathrm{pq}} \cdot m_{\mathrm{s,q}} - \mathbf{m}_{0,\mathrm{p}} \times (i\alpha\omega \mathbf{m}_{\mathrm{s,p}} - \omega_{\mathrm{J}} \mathbf{T}_{\mathrm{p}} \times \mathbf{m}_{\mathrm{s,p}}). \quad (10)$$

Here, $p, q = 1, 2$ enumerates two waveguides, and the tensor $\hat{\Omega}_{\mathrm{pq}}$ has the form,

$$\hat{\Omega}_{\mathrm{pq}} = \omega_0 \delta_{\mathrm{pq}} \hat{I} + \omega_{\mathrm{M}} \hat{F}(d_{\mathrm{pq}}) + \kappa(\delta_{\mathrm{pq}} - 1)\hat{I}. \quad (11)$$

Here, we introduce $\omega_0 = \gamma H_0 + \frac{2\gamma A_{\mathrm{ex}} k^2}{\mu_0 M_{\mathrm{s}}} + \kappa$, $\kappa = \gamma \frac{J_{\mathrm{RKKY}}}{\mu_0 M_{\mathrm{s}} t_{\mathrm{p}}}$, and $\omega_{\mathrm{M}} = \gamma M_{\mathrm{s}}$. The wave vector k is equal to $\sqrt{k_{\mathrm{x}}^2 + k_{\mathrm{y}}^2}$, the distance between the two waveguides $d_{12} = t + \sigma$, t is the waveguide thickness, and σ is the gap between the waveguides. The dynamic magnetodipolar interaction is described by the tensor $\hat{\mathbf{F}}$:[9–12]

$$\begin{aligned} \hat{F}(d_{\mathrm{pq}}) &= \int \hat{N}(d_{\mathrm{pq}}) e^{i\mathbf{k}\cdot\mathbf{r}} \frac{d^2\mathbf{k}}{(2\pi)^2}, \\ \mathbf{N}^{\alpha\beta}(d_{\mathrm{pq}}) &= \frac{1}{t} \int D_{\mathrm{p}}(k_{\mathrm{z}}) D_{\mathrm{q}}^*(k_{\mathrm{z}}) \frac{k_\alpha k_\beta}{k^2} e^{ik_{\mathrm{z}} d_{\mathrm{pq}}} \frac{dk_{\mathrm{z}}}{2\pi}. \end{aligned} \quad (12)$$

The "shape amplitude" $D_{\mathrm{p}}(k_{\mathrm{z}}) = \int_0^t m(z) e^{-ik_{\mathrm{z}} z} dz$ describes the influence of the finite thickness t of the thin waveguide. The width profile of the SW mode in the waveguide is usually nonuniform ($m(z) \sim \cos(k_{\mathrm{z}}^p z)$) due to the pinning effect from the geometric boundaries. In general, if the thickness of the waveguide is close to or smaller than the material exchange length or the effective boundary condition are free, the SW profile is almost uniform, i.e. $m(z) = 1$. Then, setting $\psi_{\mathrm{p}}^{\pm} = \delta m_{\mathrm{x,p}} \pm i\delta m_{\mathrm{z,p}}$, we obtain the spin wave equation $\omega \boldsymbol{\psi} = \hat{H} \boldsymbol{\psi}$

with $\boldsymbol{\psi} = (\psi_1^+, \psi_1^-, \psi_2^+, \psi_2^-)$ from Eq. (10), and the 4×4 Hamiltonian $\hat{H}$ is non-Hermitian,

$$\hat{H} = \begin{pmatrix} \omega_1^+ - i\omega_J^+ & \omega_x^+ - \omega_z^+ & \omega_{x,d}^+ + \omega_{z,d}^+ - \kappa^+ & \omega_{x,d}^+ - \omega_{z,d}^+ \\ -\omega_x^- + \omega_z^- & -\omega_1^- - i\omega_J^- & -\omega_{x,d}^- + \omega_{z,d}^- & -\omega_{x,d}^- - \omega_{z,d}^- + \kappa^- \\ \omega_{x,d}^+ + \omega_{z,d}^+ - \kappa^+ & \omega_{x,d}^+ - \omega_{z,d}^+ & \omega_1^+ + i\omega_J^+ & \omega_x^+ - \omega_z^+ \\ -\omega_{x,d}^- + \omega_{z,d}^- & -\omega_{x,d}^- - \omega_{z,d}^- + \kappa^- & -\omega_x^- + \omega_z^- & -\omega_1^- + i\omega_J^- \end{pmatrix} \quad (13)$$

Here, we define $\omega_1 = \omega_0 + \omega_M F^{xx}(0)/2 + \omega_M F^{zz}(0)/2$, $\omega_x = \omega_M F^{xx}(0)/2$, $\omega_z = \omega_M F^{zz}(0)/2$, $\omega_{x,d} = \omega_M F^{xx}(d_{12})/2$, $\omega_{z,d} = \omega_M F^{zz}(d_{12})/2$, $\omega_1^\pm = \omega/(1 \pm i\alpha)$, $\omega_{x(z),d}^\pm = \omega_{x(z),d}/(1 \pm i\alpha)$, $\omega_J^\pm = \omega_J/(1 \pm i\alpha)$ and $\kappa^\pm = \kappa/(1 \pm i\alpha)$.

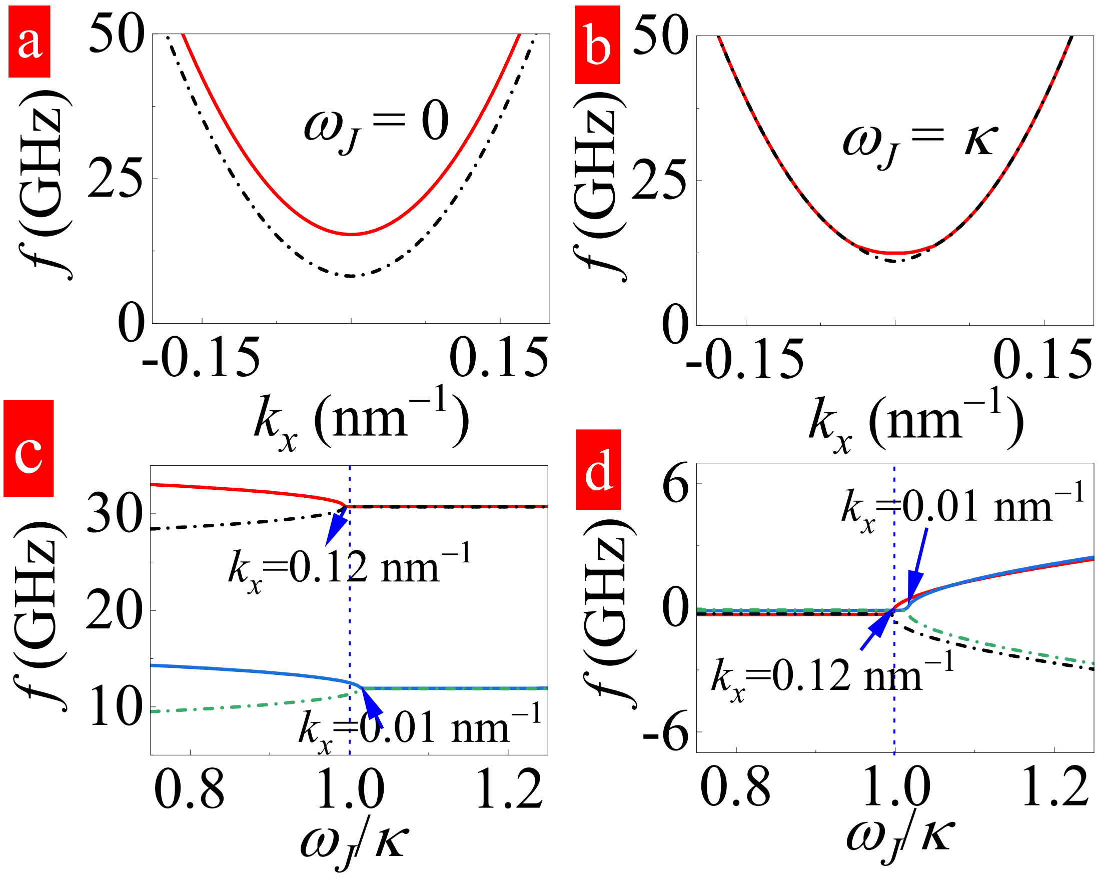

Figure 7: (a-b) Merging of the two magnon modes dispersion (a, $\omega_J = 0$) when approaching the EP (b, $\omega_J = \kappa$). (c) Real and (d) imaginary parts of the two eigenmode frequencies $f = \omega/(2\pi)$ as we scan ω_J/κ at the wave vector $k_x = 0.01$ nm^{-1} and 0.12 nm^{-1}.

Without SOT ($\omega_J = 0$), we obtain four eigenfrequencies from $\hat{H}$. Two positive frequencies

correspond to right-hand precessions around their ground states (the other two negative frequencies are for left-hand precessions). The two positive eigenfrequencies are,

$$
\begin{aligned}
(1+\alpha^2)\omega = & \sqrt{(\omega_0 \mp \kappa + 2\omega_{\mathrm{x}} \pm 2\omega_{\mathrm{x,d}})(\omega_0 \mp \kappa + 2\omega_{\mathrm{z}} \pm 2\omega_{\mathrm{z,d}}) + [i\alpha(\omega_{\mathrm{x}} - \omega_{\mathrm{z}} \pm \omega_{\mathrm{x,d}} \mp \omega_{\mathrm{z,d}})]^2} \\
& + i\alpha(\omega_0 \mp \kappa + \omega_{\mathrm{x}} + \omega_{\mathrm{z}} \pm \omega_{\mathrm{x,d}} \pm \omega_{\mathrm{z,d}}).
\end{aligned} \tag{14}
$$

With the above analytical expression, we insert the same material parameters used in the simulations, and calculate the real parts (i.e., the dispersion relations) of the eigenfrequencies, see Fig. 7. The calculated spinwave dispersion is in a good agreement with the simulation results. Scanning $\omega_{\mathrm{J}}/\kappa$, the two separated real parts of eigenfrequencies merge at the same value at the EP, and the two imaginary parts are obviously separated after EP. Comparing the calculations for different wavevectors k_{x}, the EP with a lower k_{x} has a larger EP due to the nature of the dynamic dipole-dipole interaction. The small difference between the simulated and the analytical results can be attributed to the approximations made in the analytical model.

References

(1) Ryu, J.-W.; Son, W.-S.; Hwang, D.-U.; Lee, S.-Y.; Kim, S. W. Exceptional points in coupled dissipative dynamical systems. *Phys. Rev. E* **2015**, *91*, 052910.

(2) Hodaei, H.; Hassan, A. U.; Wittek, S.; Garcia-Gracia, H.; El-Ganainy, R.; Christodoulides, D. N.; Khajavikhan, M. Enhanced sensitivity at higher-order exceptional points. *Nature* **2017**, *548*, 187–191.

(3) Demidov, V. E.; Kostylev, M. P.; Rott, K.; Münchenberger, J.; Reiss, G.; Demokritov, S. O. Excitation of short-wavelength spin waves in magnonic waveguides. *Appl. Phys. Lett.* **2011**, *99*, 082507.

(4) Wang, X.-g.; Chotorlishvili, L.; Guo, G.-h.; Jia, C.-L.; Berakdar, J. Thermally assisted skyrmion drag in a nonuniform electric field. *Phys. Rev. B* **2019**, *99*, 064426.

(5) Bruno, P. Theory of interlayer exchange interactions in magnetic multilayers. *J. Phys.: Condens. Matter* **1999**, *11*, 9403–9419.

(6) Demasius, K.-U.; Phung, T.; Zhang, W.; Hughes, B. P.; Yang, S.-H.; Kellock, A.; Han, W.; Pushp, A.; Parkin, S. S. P. Enhanced spin-orbit torques by oxygen incorporation in tungsten films. *Nat. Commun.* **2016**, *7*, 10644.

(7) Liu, L.; Moriyama, T.; Ralph, D. C.; Buhrman, R. A. Spin-Torque Ferromagnetic Resonance Induced by the Spin Hall Effect. *Phys. Rev. Lett.* **2011**, *106*, 036601.

(8) Zhang, S.; Li, Z. Roles of Nonequilibrium Conduction Electrons on the Magnetization Dynamics of Ferromagnets. *Phys. Rev. Lett.* **2004**, *93*, 127204.

(9) Wang, Q.; Pirro, P.; Verba, R.; Slavin, A.; Hillebrands, B.; Chumak, A. V. Reconfigurable nanoscale spin-wave directional coupler. *Sci. Adv.* **2018**, *4*, e1701517.

(10) Verba, R.; Melkov, G.; Tiberkevich, V.; Slavin, A. Collective spin-wave excitations in a two-dimensional array of coupled magnetic nanodots. *Phys. Rev. B* **2012**, *85*, 014427.

(11) Beleggia, M.; Tandon, S.; Zhu, Y.; Graef, M. D. On the magnetostatic interactions between nanoparticles of arbitrary shape. *J. Magn. Magn. Mater.* **2004**, *278*, 270 – 284.

(12) Wang, X.-g.; Chotorlishvili, L.; Guo, G.-h.; Berakdar, J. High-Fidelity Magnonic Gates for Surface Spin Waves. *Phys. Rev. Applied* **2019**, *12*, 034015.
